# Morphological analysis of the filum terminale and detailed description of the distal filum terminale externum: a cadaveric study

**DOI:** 10.3389/fnana.2025.1547165

**Published:** 2025-03-25

**Authors:** Edgar Buloz-Osorio, Marisa Ortega-Sánchez, Miguel B. Royo-Salvador, Alfonso Rodríguez-Baeza

**Affiliations:** ^1^Institut Guttmann, Institut Universitari de Neurorehabilitació Affiliated with the Universitat Autònoma de Barcelona, Badalona, Spain; ^2^Fundació Institut d’Investigació en Ciències de la Salut Germans Trias i Pujol, Barcelona, Spain; ^3^Human Anatomy and Embryology Unit, Department of Morphological Sciences, Faculty of Medicine, Universitat Autònoma de Barcelona, Cerdanyola del Vallès, Spain; ^4^Institute of Legal Medicine and Forensic Sciences of Catalonia, Barcelona, Spain; ^5^Institut Chiari and Siringomielia and Escoliosis de Barcelona, Barcelona, Spain

**Keywords:** filum terminale, spinal cord, cadaveric study, neuroanatomy, dura mater, conus medullaris, dural sac, tethered cord syndrome

## Abstract

This observational, descriptive anatomical cadaveric study aimed to identify, characterize, and analyze the morphometric parameters of the filum terminale (FT) and macroscopically describe the distal insertion of the FTE. The FT is a complex, three-dimensional, fibro-cellular structure of connective tissue and glial cells, extending from the conus medullaris (CM) to the dural sac (DS) and coccyx. It is divided into two segments: an intradural filum terminale internum (FTI) and extradural filum terminale externum (FTE). Few studies have comprehensively addressed its morphometric characteristics in the last decades. Thirty-eight embalmed (M = 16, F = 22) human cadavers were examined to determine the CM-FTI and DS-FTE vertebral levels and FT, FTI, and FTE lengths and widths. FTI and FTE segmental diameters, correlations, gross characteristics, tension, and mobility *in situ* and *ex vivo* were assessed. FTE distal insertion is thoroughly described. FT, FTI, and FTE mean lengths were 254.32 mm (±26.46), 152.75 mm (±22.02), and 106.64 mm (±12.21), respectively. The CM-FTI junction was observed at the L1-L2 disk space (32.1%), DS-FTE fusion in the upper third of S2 (39.3%), and FTI-DS fusion in the DS midline (46.4%). FT length and FTI, FTE lengths were directly correlated, as were all FTI diameters. FT gross characteristics were an irregular surface (71.4%), bright hue (57.1%), macroscopic FTI-CM contrast (64.3%), filiform shape (60.7%), and movement-resistance (53.6%). The FTE exhibited a flattened shape (64.3%), immobility (60.7%), distal insertion at Cx1 (67.8%) and one distal strand (60.7%). FTI segments ≥ 2 mm were uncommon (21.4%). The FTE distal insertion is variable, inserting as strands, with vascular tissue surrounding it. A distal coccygeal venous plexus and single or multiple strand-like insertions of the distal FTE are for the first time described in detail. Discrepancies in the morphometric parameters of the FT between studies highlight the need for standardized protocols, especially given the FT’s anatomic-clinical importance and potential role as a neural progenitor niche. We provide a comprehensive basis for future standardized morphometric analyses, acknowledging the limitations of embalmed cadaveric studies.

## 1 Introduction

**FIGURE 1:**
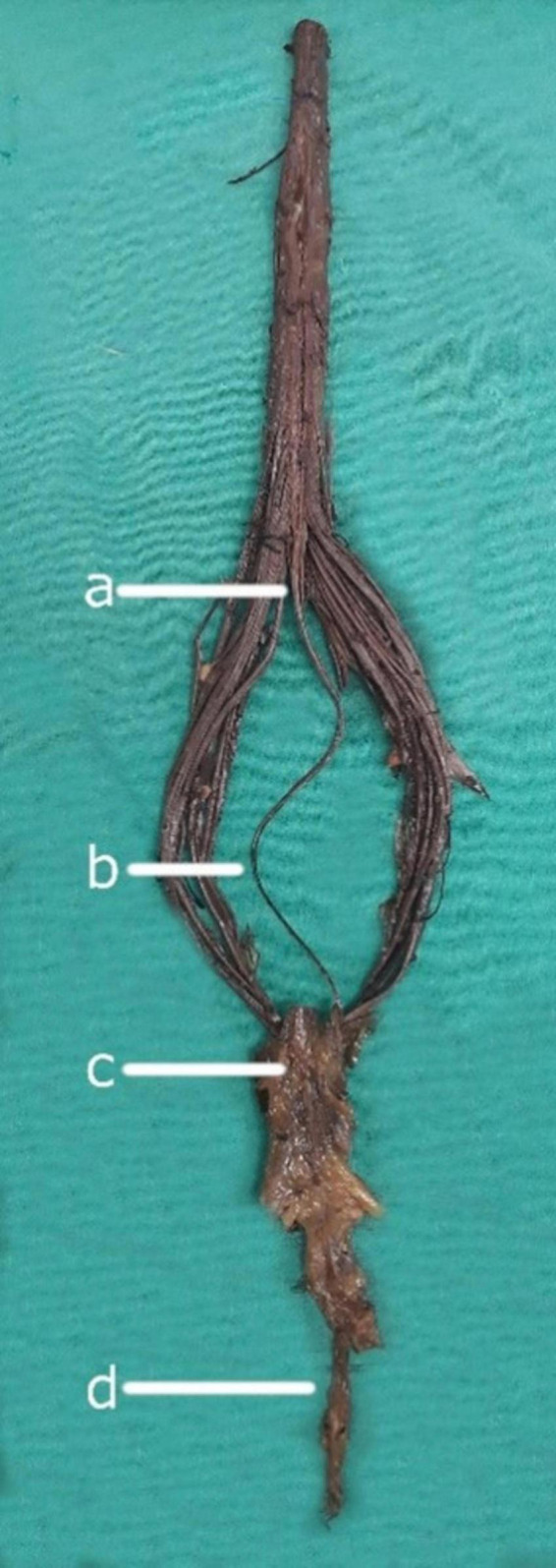
Neuroanatomical components of the filum terminale. **(a)** Conus medullaris: The tapered, distal portion of the spinal cord. **(b)** Filum terminale internum: The intradural continuation of the filum terminale, extending from the conus medullaris. **(c)** Dural sac: The membranous sheath formed by the dura mater surrounding the spinal cord and its roots. **(d)** Filum terminale externum: The portion of the filum terminale extending from the dural sac to its attachment at the coccyx.

**FIGURE 2:**
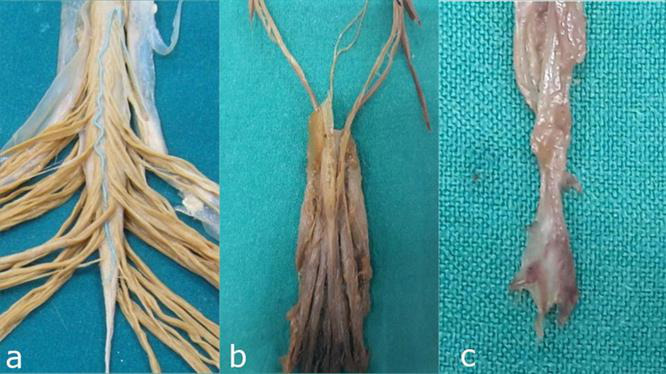
Detailed anatomical relationships and segmental anatomy of the filum terminale. **(a)** Junction of the conus medullaris and filum terminale internum: The anatomical transition between the spinal cord and its terminal filum internum. **(b)** Dural sac: The continuation of the dura mater that encases the spinal cord and extends to the filum terminale internum. **(c)** Distal segment of the filum terminale externum: The extrameningeal portion of the filum terminale, passing beyond the dural sac to attach at the coccyx.

The filum terminale (FT) is a complex, three-dimensional fibrous band of connective tissue that extends from the distal part of the conus medullaris (CM) to the coccyx (Abdulrazeq et al., 2023; De Vloo et al., 2016; Hansasuta et al., 1999; Pinto et al., 2002; Standring, 2021). It is traditionally classified into two distinct segments: the intradural part = filum terminale internum (FTI); and the extradural part = filum terminale externum (FTE) or coccygeal ligament (Dauber et al., 2021; Pinto et al., 2002; Saker et al., 2017; Figures 1, 2). The FT is primarily composed of collagen, elastic fibers, glial cells, and blood vessels (Li et al., 2016; Nasr et al., 2018).

Macroscopically, the FT is described as a slender, thin, filiform, threadlike filament, extending caudally from the CM, the caudal termination of the spinal cord (Rouvière et al., 2009; Standring, 2021). The FTI continues from the CM to the inferior dural sac (DS), to which it fuses, forming the FTE. It continues from the distal portion of the DS and adheres or fuses into the dorsal coccygeal periosteum, typically at the first coccygeal vertebra (Cx1), occasionally reaching the second coccygeal vertebra (Cx2) (Dauber et al., 2021; De Vloo et al., 2016; Saker et al., 2017). While the CM-FTI junction is commonly located at the first lumbar vertebra (L1), reported variations include its presence in the lower third of T11 or even the upper third of L3 (De Vloo et al., C; Pinto et al., 2002).

During its descent from the CM to the DS, the FTI is surrounded by the cauda equina (CE) and extensions of the dura mater and arachnoid meninges (Namba, 2016; Standring, 2021). It continues from the CM as a long, slender, filamentous prolongation of the pia mater, inserting into the distal midline of the dura mater, which forms a cul-de-sac called the thecal or dural sac (Hansasuta et al., 1999; Rouvière et al., 2009; Standring, 2021; Tubbs et al., 2016). The DS is typically located at the superior, middle, or lower third of the second or third sacral vertebrae, marking the distal limit of the FTI (Rouvière et al., 2009; Tubbs et al., 2016). The FTI resides within the superior sacral canal, alongside the meninges and CE (De Vloo et al., 2016; Hansasuta et al., 1999; Standring, 2021).

The FTE, an extension of the dura mater, begins distally after the FTI perforates or fuses with the DS, either to its midline or lateral areas, descending through the sacral canal and emerging below the sacral hiatus. It then traverses the body of the fifth sacral vertebra and sacrococcygeal joint and terminates at the dorsum of the first or second coccygeal vertebra (Hansasuta et al., 1999; Standring, 2021; Tubbs et al., 2016). The diameter of the FT varies between humans and vertebral segments, with Yamada et al. (2007) suggesting a normal diameter of 1.50 mm ± 0.50 mm.

The vascular anatomy of the FT, often overlooked, includes the single filum terminale artery as a continuation of the ventral spinal artery, although individuals with a duplicate FT artery have been reported (Djindjian et al., 1988; Harmeier, 1933; Iampreechakul et al., 2022; Lim et al., 2011). The filum terminale artery contributes to the anterior spinal arterial axis and its vascular basket as a distal branch (Rojas et al., 2018). It has also been described as a ventral extension of the descending branch of the artery of Adamkiewicz (Djindjian et al., 1988; Lim et al., 2011; Schmolling et al., 2023). At the most distal segment of the CM, this artery may bifurcate or trifurcate, forming anastomotic connections with posterior spinal arteries (Lim et al., 2011). It irrigates the FT body and decreases its diameter along its longitudinal course (Lim et al., 2011; Tubbs et al., 2016). A small artery supplying the coccygeal nerve, adhering to the proximal portion of the FT, has also been reported (Djindjian et al., 1988).

The filum terminale vein typically lies dorsal to the artery and extends from the most distal part of the FTE to the most proximal segments of the FTI and CM (Lim et al., 2011; Rouvière et al., 2009; Standring, 2021). The FT vein and ventral veins of the spinal cord are connected through the sacral extradural venous plexus (Djindjian et al., 1988).

Research on the morphometric parameters of the FT is scarce, with only six studies dedicated exclusively to the anatomical characteristics of the FT published over the last decades (Cai et al., 2024; De Vloo et al., 2016; Fontes et al., 2006; Nasr et al., 2018; Picart et al., 2019; Pinto et al., 2002). Studies differed greatly in the type of cadaver evaluated (fresh, frozen, or embalmed), in the selection of different FTI and FTE lengths for segmental and width analysis, and in the aim and methodology of the studies. These discrepancies have remained unsolved, mostly due to methodological limitations, such as the lack of standardized analysis protocols.

To the best of our knowledge, the distal insertion of the FTE has not been significantly described in the existing literature.

Recent attention has been paid to it given its clinical implications in spinal pathologies like scoliosis (Fluss et al., 2024), tethered cord syndrome (Chern et al., 2011; Miyagami et al., 2024; Otto et al., 2023; Yamada et al., 2007), filum disease syndrome (Royo-Salvador et al., 2020; Royo-Salvador et al., 2024), myxopapillary ependymomas (Balodis et al., 2024; Kouhen et al., 2024), neuroendocrine tumors (Dharanipathy et al., 2023; Popov et al., 2023; Zhu et al., 2023), and arteriovenous fistulas (Guo and Yu, 2023; Iampreechakul et al., 2022; Ota, 2023; Schmolling et al., 2023; Zhu et al., 2023). In addition, the FT has been proposed as an atypical neural progenitor niche in humans and animals (Arvidsson et al., 2011; Chrenek et al., 2017; Jiang et al., 2022; Klinge et al., 2022; Nakano et al., 2019; Varghese et al., 2010). This generates a need for better and more thorough studies of the FT due to the knowledge gap between the anatomical characteristics of the structure and its clinical significance.

The current study aimed to identify, characterize, and analyze the structure, morphometric variables, parameters, and gross features of the FT. We also sought to describe novel macroscopic characteristics of the distal insertion of the FTE. Furthermore, we intended to present a consensus protocol for morphometric and descriptive analysis of the FT, with the aim of standardizing the approach to studying its anatomy, biomechanical properties, and underestimated clinical-surgical significance.

## 2 Materials and methods

### 2.1 Ethical procedures

All cadavers were donated for medical and scientific research purposes, and informed consent was obtained from donors or their legal representatives. Ethics approval for this study was granted by the Human Experimentation Ethics Committee of the Universitat Autònoma de Barcelona (Procedure 2904, approved on 03/27/2015).

### 2.2 Selection of specimens

Thirty-eight human spinal cords obtained from embalmed cadavers were used in this study. Embalmed cadavers were used in the study due to their availability and accessibility within our department. Additionally, the stability and consistency of the anatomical structures, along with the prevention of postmortem changes, were key factors in selecting this type of specimen. Specimens exhibiting spinal pathologies were excluded from this study.

### 2.3 Dissection process

The dissection procedure was conducted with the cadaver in a prone position. A midline dorsal skin and aponeurotic incision was made above the thoracic and lumbar spinal processes, extending to the median sacral crests and coccyx. Following exposure of the lumbar laminae, extensive bilateral lumbosacral laminectomies spanning from T11 to S3–S5 were performed. Subsequently, a midline longitudinal incision was made through the caudal dura mater to expose the CM and FTI within the CM-FTI junction.

### 2.4 Anatomical measurements and morphological assessment

**FIGURE 3:**
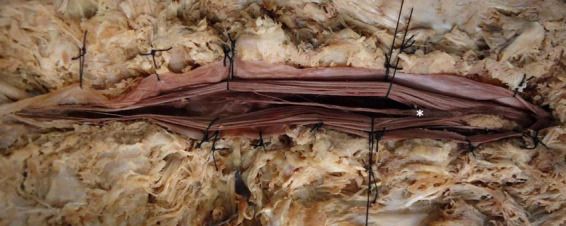
Dissection and exposure of the conus medullaris and filum terminale internum. Illustrative dissection highlighting the conus medullaris (*) and the closely associated filum terminale internum, demonstrating the spinal cord’s terminal portion and its intradural extension.

**FIGURE 4:**
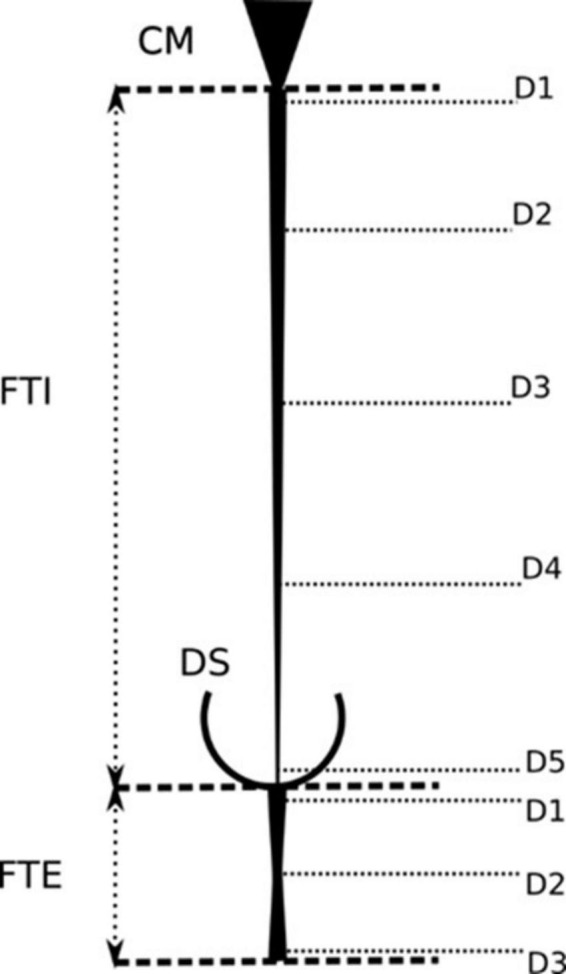
Morphometric parameters for the measurement of the filum terminale. Schematic representation of the anatomical landmarks and measurement protocols for assessing the length, width, and cross-sectional diameter of the filum terminale. These parameters are crucial for understanding its anatomical variation.

The CM-FTI junction and DS—FTE vertebral fusion levels were described following the method developed by Hansasuta et al. (1999). Lateral fixation of the dura mater and bundling of the cauda equina facilitated the visualization and description of gross anatomical features (Figure 3). *Ex vivo* measurements of FT, FTI, and FTE lengths (L) were performed, while FTI (FTI-D1 to D5) and FTE (FTE-D1 to D3) diameters (D) were assessed following the protocol described by De Vloo et al. (2016), measuring its width at five equidistant segments for FTI, and three equidistant segments for FTE (Figure 4). The biomechanical characteristics of the FTI and FTE, such as tension and mobility, were evaluated *in situ*, without external resistance, the structure was characterized as either mobile or immobile based on manual traction assessment.

### 2.5 Filum terminale externum assessment

Macroscopic evaluation of the distal insertion of the FTE and its surrounding tissue was performed, both *in situ* and *ex vivo*, and describing the number of strands by superimposing photographic samples of the FTE strands.

### 2.6 Equipment and instruments

Measurements were taken using a 300 mm high-precision digital Vernier caliper (Würth, Künzelsau-Gaisbach, Germany). Statistical analysis, including calculation of means and standard deviations for continuous variables and frequencies and percentages for nominal variables, were conducted using GraphPad Prism v8.00 (GraphPad Software, La Jolla, California, USA).

### 2.7 Statistical analysis

Independent *t*-tests and one-way analysis of variance (ANOVA) were employed to identify relationships between means, while a two-tailed bivariate Pearson’s *r* correlation coefficient test and Spearman’s ρ were employed to identify correlations between continuous variables and between ordinal variables, respectively. Statistical significance was set at *p* ≤ 0.05.

## 3 Results

### 3.1 Demographics

The study analyzed thirty-eight human embalmed cadavers, with a mean age at death of 82.1 years (SD 8.7, range 60–94 years), comprised of twenty-two females (60.7%) and sixteen males (39.3%). Age was not significantly correlated with the total FT or FTI lengths. However, a small inverse correlation was observed between age and FTE length (*p* = 0.040; *r* = −0.390).

### 3.2 CM-FTI and DS-FTE junctions

Predominantly, the CM-FTI junction occurred at the L1-L2 disk space (32.1%), while the distal DS-FTE junction was predominantly localized in the upper third of S2 (39.3%) (Table 1). FTI fusion within the DS was most often observed in the midline (46.4%) (Table 2). Males and females were not significantly different in their CM-FTI junction (*p* = 0.087) or FTI-DS fusion levels (*p* = 0.431).

**TABLE 1:** Literature comparing conus medullaris and dural sac vertebral levels.

References	Level of conus medullaris	Level of dural sac
Current study	L1/L2 disc	Upper S2
Picart et al., 2019	Lower L1 and L1/L2 disc	Lower S2
Pokanan et al., 2020	–	S1/S2 disc
Nasr et al., 2018	Lower L1	Upper S2
De Vloo et al., 2016	Mid L1	Mid S2
Pinto et al., 2002	Mid L1	Upper S2
Hansasuta et al., 1999	–	S1/S2 disc

**TABLE 2:** Literature comparing midline and lateral filum terminale internum fusion in the dural sac.

References	FTI insertion left	FTI insertion midline	FTI insertion right
Current study	42.8%	46.4%	10.7%
Picart et al., 2019	10.0%	80.0%	10.0%
Hansasuta et al., 1999	3.7%	88.9%	7.4%

FTI, filum terminale internum.

### 3.3 Anatomical measurements and correlations

The FT and FTI lengths were significantly greater in male specimens (*p* = 0.002) than in female specimens, but the same was not true of the FTE lengths (*p* = 0.476). Table 3 lists the mean total lengths and segmental diameters of the FT, FTI, and FTE. Direct correlation was noted between the total FT and FTI lengths (*p* < 0.001, *r* = 0.889), with a moderate direct correlation observed between FT and FTE lengths (*p* = 0.002; *r* = 0.559). Conversely, no significant correlation was found between the FTI and FTE lengths (*p* = 0.556). Furthermore, a small direct correlation was identified between FT lengths and FTI-D4 (*p* = 0.019; *r* = 0.441) (*p* = 0.0.03; *r* = 0.405) (Table 4).

**TABLE 3:** Literature comparing types of specimen, numbers of cadavers, and segmental and total filum terminale lengths and diameters.

References	Cadaver	*N*	FT-L	FTI-L	FTE-L	FTI-D1	FTI-D2	FTI-D3	FTI-D4	FTI-D5	FTE-D1	FTE-D2	FTE-D3
Current study	E		254.32 ± 26.46	152.75 ± 22.02	106.64 ± 12.21	1.66 ± 0.38	1.24 ± 0.28	0.90 ± 0.37	0.83 ± 0.34	1.12 ± 0.31	1.66 ± 0.30	1.32 ± 0.22	1.49 ± 0.51
Picart et al., 2019	F	10	–	167.13 ± 21.69	87.59 ± 17.46	1.84 ± 0.34	0.88 ± 0.20	0.71 ± 0.22	0.67 ± 0.30	0.74 ± 0.24	1.05 ± 0.38	0.77 ± 0.22	0.59 ± 0.24
Nasr et al., 2018	E	25	202.9 ± 20.9	162.3 ± 12.8	74.8 ± 8.1	1.7 ± 0.2	–	0.74 ± 0.14	–	–	–	0.48 ± 0.14	–
De Vloo et al., 2016	F and E	20	–	160.01	69.86	1.91	0.92	0.8	0.68	0.68	1.07	0.73	1.02
Fontes et al., 2006	F	20	–	155.4	–	1.56 (0.31–2.30)	–	1.03 (0.14–2.20)	–	–	–	–	–
Tubbs et al., 2005	E	15	–	–	80 (70–105)	–	–	–	–	–	–	–	–
Pinto et al., 2002	F	41	–	156.44 (112.8–211.1)	–	1.38 (0.4–2.5)	–	0.76 (0.1–1.55)	–	–	–	–	–

E, embalmed; F, fresh; FT, filum terminale; FTI, filum terminale internum; FTE, filum terminale externum, L, length; D, diameter; D1 to D5, proximal-to-distal segmental diameters of FTI and FTE; CM, conus medullaris; DS, dural sac.

**TABLE 4:** Correlation matrix analysis of the filum terminale (FT), filum terminale internum (FTI) and filum terminale externum (FTE) lengths (L) and diameters (D).

	FT length	FTI length	FTE length	FTI-D1	FTI-D2	FTI-D3	FTI-D4	FTI-D5	FTE-D1	FTE-D2	FTE-D3
FT length	**1**	**0.889** ***	**0.559** **	0.056	0.122	0.284	**0.441** *	0.008	-0.097	-0.137	-0.023
FTI length	**0.889** ***	**1**	0.116	0.053	-0.036	0.214	**0.405** *	-0.082	-0.224	-0.273	0.028
FTE length	**0.559** **	0.116	**1**	0.028	0.333	0.233	0.225	0.17	0.201	0.198	-0.100
FTI-D1	0.056	0.053	0.028	**1**	**0.664** ***	**0.424** *	**0.428** *	**0.478** *	0.078	-0.208	**0.551** **
FTI-D2	0.122	-0.036	0.333	**0.664** ***	**1**	**0.681** ***	**0.600** ***	0.371	0.207	-0.036	0.328
FTI-D3	0.284	0.214	0.233	**0.424** *	**0.681** ***	**1**	**0.854** ***	**0.498** **	0.051	-0.055	0.187
FTI-D4	**0.441** *	**0.405** *	0.225	**0.428** *	**0.600** ***	**0.854** ***	**1**	**0.586** ***	-0.015	-0.22	0.094
FTI-D5	0.008	-0.082	0.17	**0.478** *	0.371	**0.498** **	**0.586** ***	**1**	-0.15	**−0.399** *	-0.11
FTE-D1	-0.097	-0.224	0.201	0.078	0.207	0.051	-0.015	-0.15	**1**	**0.733** ***	0.303
FTE-D2	-0.137	-0.273	0.198	-0.208	-0.036	-0.055	-0.22	**−0.399** *	**0.733** ***	**1**	0.288
FTE-D3	-0.023	0.028	-0.100	**0.551** **	0.328	0.187	0.094	-0.11	0.303	0.288	**1**

FT, filum terminale; FTI, filum terminale internum; FTE, filum terminale externum; D, diameter; D1–D5, proximal-to-distal segmental diameters of FTI and FTE; CM, conus medullaris; DS, dural sac. The bold values to indicate *p < 0.05, **p < 0.005, ***p < 0.001. [Based on the model developed by De Vloo et al. (2016)].

Significant associations were found between the FTI’s most proximal diameter (FTI-D1) and all other FTI diameters. Inter-diameter associations within the FTI were common, with significant positive correlations. Furthermore, a strong direct correlation was observed between the FTE-D1 and FTE-D2 levels (*p* < 0.001, ρ 0.733) (Table 4).

### 3.4 FTI diameters and macroscopic features

Six specimens (15.7%) exhibited FTI diameters exceeding 2 mm (range: 2.01 mm–8.28 mm), with one case coinciding with low-level sacral dysmorphism at S3. Macroscopic features of the FTI and FTE are described in Table 5.

**TABLE 5:** Macroscopic features of the filum terminale.

Shape & surface	Results	Gross aspects, contrast & tension	Results	FTE characteristics	Results
**FTI shape**	Filiform 60.71% ribbed 32.14% flattened 7.14%	**FT gross aspects**	Bright 57.14% opaque 42.86%	**FTE mobility**	No 60.71% Yes 39.29%
**FTE shape**	Flattened 64.29% filiform 35.71%	**FTI contrast from CM**	No 64.29% Yes 35.71%	**Distal FTE insertion**	Cx1 67.86% Cx2 32.14%
**FT surface**	Irregular 71.43% regular 28.57%	**FTI tension**	No 53.57% Yes 46.43%	**Number of FTE distal strands**	“1” (60.71%) “2” (17.86%) “3” (7.14%) “4” (14.29%)

FT, filum terminale; FTI, filum terminale internum; FTE, filum terminale externum; CM, conus medullaris.

### 3.5 FTE observations

**FIGURE 5:**
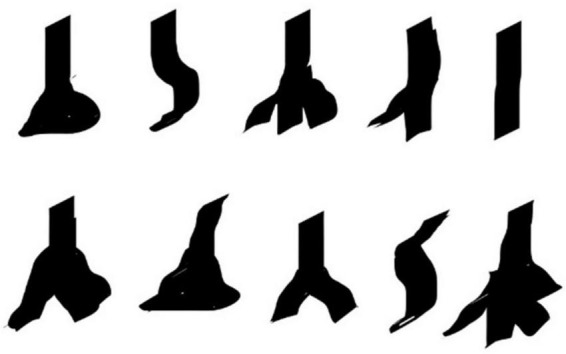
Macroscopical variability in the periosteal attachment of the filum terminale externum. Silhouettes illustrating the different forms of filum terminale externum insertion at the coccyx.

**FIGURE 6:**
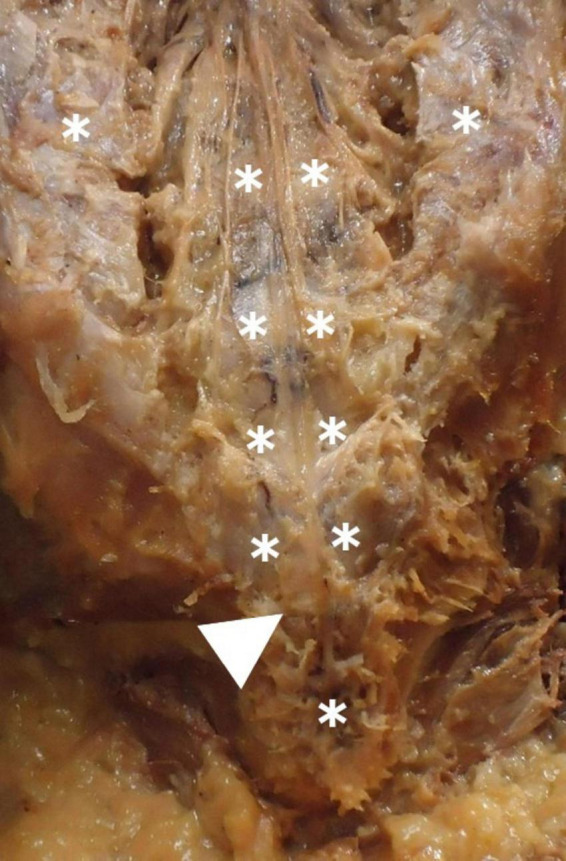
Periosteal insertion of the filum terminale externum at the dorsal coccyx and associated vascular structures. Shows the periosteal attachment of the filum terminale externum at the dorsal coccyx (arrow), highlighting the surrounding venous plexus (*). The vascular structures in this region may influence surgical approaches and pathology.

Macroscopically, the distal periosteal insertion of the FTE had diverse shapes, typically manifesting as a pyramid (Figure 5). No correlation was found between FTE lengths and the number of distal FTE strands (*p* = 0.177, *r* = −0.26). Vascular tissue was macroscopically observed superficially and surrounding the distal FTE insertion in 71.4% of specimens, predominantly resembling venous tissue (Figure 6). Anatomical scrutiny revealed no neural or bone-related variations, and fatty fila remained absent throughout all specimens.

## 4 Discussion

### 4.1 Bridging the gap

The filum terminale (FT) has traditionally been overlooked in neuroanatomical studies, despite its clinical relevance. The prevalence of FT-related syndromes and diseases, such as filum terminale lipomas, Tethered Cord Syndrome (TCS), arteriovenous fistulas (AVFs), filar cysts, paragangliomas, epidermoid cysts, hemangioblastomas, thickened filum terminale, duplicate filum terminale, and Tarlov cysts, remains largely unknown and is likely underappreciated; recently, there has been increasing interest in analyzing its morphological characteristics, due to its implications for spinal pathologies (Royo-Salvador et al., 2020; Yamada et al., 2004; Yamada, 2016) and caudal anesthesia (Namba, 2016), and its potential role as a neural progenitor cell niche (Chrenek et al., 2017). However, gross anatomical descriptions of the FT are sparse and seldom detailed, necessitating the integration of various parameters and variables from previous studies (De Vloo et al., 2016; Fontes et al., 2006; Hansasuta et al., 1999; Nasr et al., 2018; Picart et al., 2019; Pinto et al., 2002) to conduct a comprehensive analysis and propose a consensus protocol for describing the FT (Supplementary Table 1).

### 4.2 Our study and prior research

**FIGURE 7:**
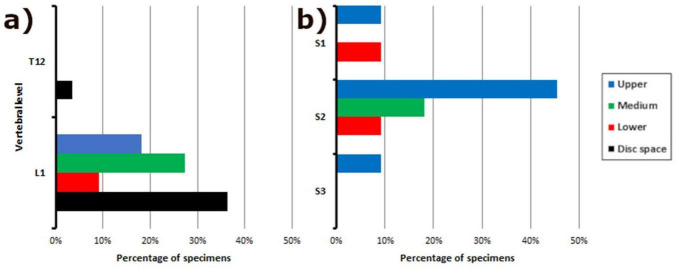
Frequency distribution of vertebral levels in relation to filum terminale anatomy. Bar graph illustrating the distribution of vertebral levels associated with: **(a)** The junction of the conus medullaris and filum terminale internum; **(b)** The fusion between the dural sac and the filum terminale externum. This data provides insight into the variability of these key anatomical landmarks along the spine.

Our morphometric and descriptive data align with some of the findings from prior studies (De Vloo et al., 2016; Hansasuta et al., 1999; Nasr et al., 2018; Picart et al., 2019; Pinto et al., 2002; Pokanan et al., 2020). Although the CM tends to be at L1, discrepancies and variations exist in its specific localization. While our findings and those of Picart et al. (2019) indicate that the CM is located at the disk space between L1 and L2, other authors (De Vloo et al., 2016; Nasr et al., 2018; Pinto et al., 2002) describe it superiorly. The DS is classically described as being located at S2. In our study, it was located in the upper third of S2, as previously reported (Hansasuta et al., 1999; Nasr et al., 2018; Pinto et al., 2002; Pokanan et al., 2020), but also at S1 and S3 (Figure 7).

Few studies have described FTI-DS fusion in detail. Hansasuta et al. (1999) and Picart et al. (2019) observed FTI-DS fusion at the midline of the DS, but we found that most of the FTI fuses not only in the midline but also in a lateral position. Interestingly, we found that our mean FTI lengths were smaller (De Vloo et al., 2016; Fontes et al., 2006; Nasr et al., 2018; Picart et al., 2019; Pinto et al., 2002) and our FTE lengths were larger than those reported for other studies, but within the general range of prior studies (De Vloo et al., 2016; Nasr et al., 2018; Picart et al., 2019; Tubbs et al., 2005). Some of these differences may be explained by the measurement techniques or different preservation processes applied. Pinto et al. (2002) advocate studying fresh cadavers to avoid possible distortions caused by the embalming process, although De Vloo et al. (2016) stated that the results observed from fresh and embalmed cadavers may be comparable.

Consistent with the results of other studies (De Vloo et al., 2016; Picart et al., 2019; Pinto et al., 2002), we failed to identify any significant correlation between FTE lengths and DS levels. Our results yielded similar conclusions to previous studies (De Vloo et al., 2016; Picart et al., 2019), as we discovered no correlation between FTI lengths and FTE lengths. Several authors (De Vloo et al., 2016; Fontes et al., 2006; Nasr et al., 2018; Picart et al., 2019) have reported similar values to our study for all FTI diameters. We identified strong correlations between FTI-D1 and all the other FTI diameters and but no between FTE diameters except for proximal and middle FTE diameters; therefore, the first segment of the FTI can be used to predict the width of other FTI diameters.

### 4.3 Main findings

The FTI is described as having a pyramidal shape, and the FTE an hourglass shape; but these are not entirely accurate descriptions. Our evidence shows that the distal FTI diameter may be larger at a point between the midpoint and its fusion with the DS, where it becomes wider caudally. The proximal FTE diameter is larger than the distal FTI diameter, indicating that the FT widens before and after the FTI-DS fusion, a characteristic that, to the best of our knowledge, has not been described before.

We decided to re-analyze the macroscopic characteristics of the complete FT for our study, examining shape, surface, contrast between CM and FTI, as well as mobility, and tension (Picart et al., 2019). Our findings do not agree with those of other studies (De Vloo et al., 2016), in that we found the FT to not have a brighter tone than the surrounding CE roots. Additionally, we found that the FTI tended to be more irregularly shaped than the FTE. The gross shapes of the FTI and FTE are substantially different, with the FTI more thread-like or filiform, while most of the FTE were flatter in most specimens (although the biomechanical relevance of this has yet to be determined).

### 4.4 The “unexplored” FTE

The distal insertion of the FTE has been historically described as occurring in the coccygeal periosteum. Very few authors even mention that it occurs specifically at Cx1 (Picart et al., 2019). However, we found distal insertion for some FTE specimens into Cx2. The FTE insertion was morphologically variable. We observed from one to four FTE strands inserted into the coccygeal periosteum, sometimes in bulk form, as a single thick strand, a Y-shape, or even triangular or pyramid-like, as described by Picart et al. (2019). A small venous plexus (Figure 6) may accompany the FTE, mostly in the distal part, at its insertion into the coccygeal periosteum. This has not been described previously in the literature, nor has any venous plexus been reported to be related exclusively to this area, or its anatomical relevance explained.

### 4.5 Operational considerations

The differences between FT parameters in fresh and embalmed cadavers have not been studied or compared extensively, and we suggest that this is an important question to address; while age may influence FT length comparisons, its effect on the measured parameters in human adults also remains unclear. Variations in measurement techniques and preservation methods may contribute to discrepancies in results; however, the available evidence remains inconclusive. While some studies have reported differences, these inconsistencies are not consistently observed across all measurements, as demonstrated in the present study.

The prone position of the cadaver during *in situ* measurements, the differences between *in situ* and *ex vivo* evaluations, and difficulties determining–microscopically and macroscopically–the end of the CM and beginning of the FTI (CM-FTI), the end of the FTE as it fuses with the DS (DS-FTE), and the beginning of the FTE all are parameters to consider. Difficulty dissecting the complete FTE, as it may adhere to the sacral dorsum, is an often-overlooked issue in FT dissection techniques.

### 4.6 Recommendations

**FIGURE 8:**
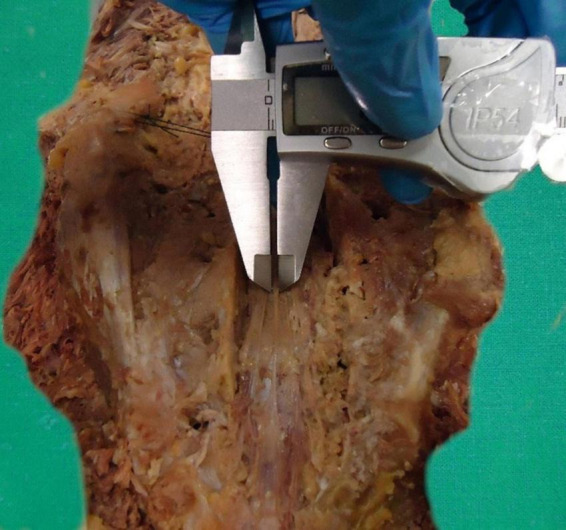
Caliper-based measurement of the diameters of the filum terminale externum. Detailed illustration of the use of calipers to measure the diameters of the filum terminale externum.

One of our objectives was to establish a basic protocol for future morphometric cadaveric studies of the FT (Supplementary Table 1). We suggest measuring the FT and to determine the CM-FTI and DS vertebral segments *in situ*, according to the method described by Hansasuta et al. (1999). We recommend characterizing the gross aspects, shape, surface, tension and mobility, number of FTE distal strands, and the FTE distal periosteal insertion *in situ* as per our protocol. *In situ* and *ex vivo* measurements of FT, FTI, and FTE lengths, and FTI and FTE diameters, as described by De Vloo et al. (2016), are also recommended to be part of this protocol (Figures 4, 8). These guidelines should facilitate a more uniform approach and provide comparable morphological data for this complex structure. A deeper understanding of the filum terminale (FT) and its anatomical variations has the potential to significantly influence clinical practices and surgical techniques, particularly in neurosurgery and spine-related interventions.

### 4.7 Future

Future research efforts should prioritize comprehensive morphological, gross macroscopic, and histological descriptions of the FT to standardize dissection techniques in cadaver specimens. Evaluation parameters in *ex vivo* and *in situ* settings, whether in fresh or embalmed cadavers, should be standardized to ensure consistency and reliability across studies. A discussion on embalming effects could mitigate potential biases. Linking anatomical variations to clinical or biomechanical outcomes could enhance the relevance of findings. The adoption and validation of this proposed standardized morphometric protocol could strengthen its impact. Imaging and *in vivo* studies could significantly enhance our understanding of the FT and its anatomical variability.

### 4.8 Limitations

This cadaveric study has several limitations, including a modest sample size, which may not fully capture anatomical variability, and the use of elderly embalmed cadavers, which might not accurately represent the morphometric characteristics of other populations. Additionally, embalming may cause tissue shrinkage compared to fresh specimens, potentially affecting measurements. We should also consider the duration of time between the embalming process and evaluation, cadaver positioning, and inherent difficulties trying to replicate living biomechanical and functional characteristics. Fresh cadaver data or a more detailed discussion on embalming effects could mitigate potential biases in future studies. The current challenge is to effectively correlate anatomical finding with medical and surgical pathologies.

## 5 Conclusion and the importance of studying the filum terminale

The FT, an often-overshadowed segment in spinal cord studies, exhibits greater variability than previously acknowledged. The vascular aspects of FT have largely been overlooked and warrant further examination. To our knowledge, this is the first paper in which a venous plexus in the most distal part of the FTE, the coccygeal vertebrae, has been described. Additionally in this paper, previously undocumented characteristics of FTE coccygeal insertion have been detailed.

Due to considerable variations in the methods used to dissect the FT, we propose a dissection protocol for evaluating FT, laying the groundwork for future investigations. This is particularly important, given the clinical relevance of the FT in spinal pathologies like tethered cord syndrome, neurovascular disorders, and its potential role as an atypical neural progenitor niche. Further study of the filum terminale is warranted, given the unresolved questions stemming from variability in previous research and its critical relevance across diverse clinical contexts, particularly for spinal surgeons, neurologists, and other specialists managing FT-related pathologies. It is our hope that this study contributes to a deeper understanding of the FT and its clinical implications, and narrows the gap between understudied anatomical segments, like the filum terminale, and clinical practice and patient care.

## Data Availability

The raw data supporting the conclusions of this article will be made available by the authors, without undue reservation.
