# Supplementary material for: Morphological analysis of the filum terminale and detailed description of the distal filum terminale externum: a cadaveric study
Source: Front Neuroanat. 2025 Mar 25;19:1547165. doi: 10.3389/fnana.2025.1547165 (PMC11975916; doi:10.3389/fnana.2025.1547165)

# Protocol & Data Collection Sheet

| **N°** |  |
| --- | --- |
| **Age** |  |
| **Gender (Male / Female)** |  |
| **Conus medullaris level (Vertebrae & Upper / Middle / Lower / Disc space)** |  |
| **Dural sac level (Vertebrae & Upper / Middle / Lower / Disc space)** |  |
| **FTI insertion / DS (Right / Center / Left)** |  |
| **FTI-L (mm)** |  |
| **FTE-L (mm)** |  |
| **FT-L (mm)** |  |
| **FTI-D1 (mm)** |  |
| **FTI-D2 (mm)** |  |
| **FTI-D3 (mm)** |  |
| **FTI-D4 (mm)** |  |
| **FTI-D5 (mm)** |  |
| **FTE-D1 (mm)** |  |
| **FTE-D2 (mm)** |  |
| **FTE-D3 (mm)** |  |
| **FTI shape (Filiform / Ribbed / Flattened)** |  |
| **FTE shape (Filiform / Ribbed / Flattened)** |  |
| **FT surface (Regular / Irregular)** |  |
| **FT gross aspect (Bright / Opaque)** |  |
| **FTI contrast from CM (Yes / No)** |  |
| **FTI tension (Yes / No)** |  |
| **FTE mobility (Yes / No)** |  |
| **Distal FTE insertion (Cx1 / Cx2 / Other)** |  |
| **# of FTE distal strands** |  |
| **Others: Fatty fillum, vascular, tumors, etc.** |  |


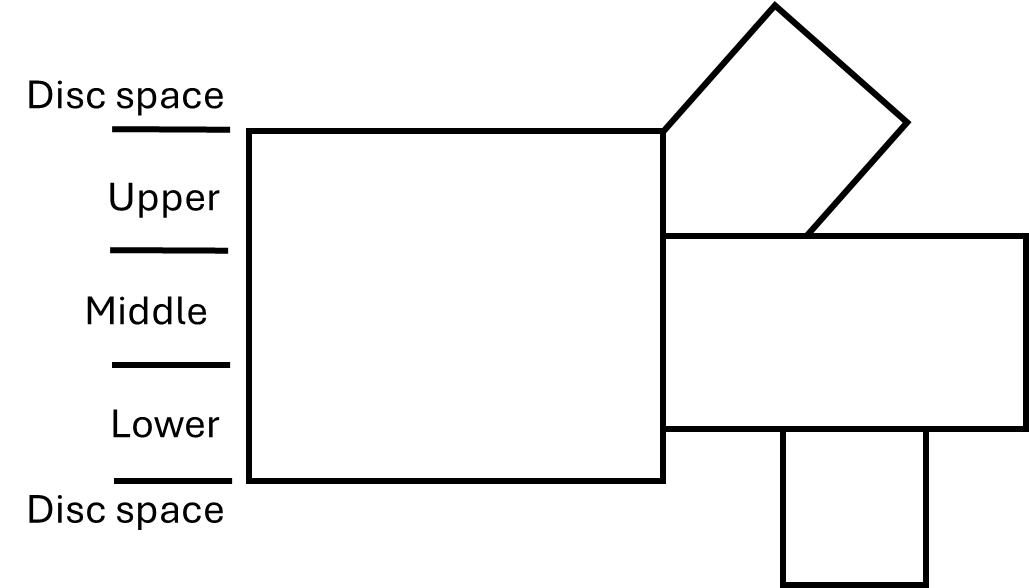

Supplement: Supplementary file 1 [file Table_1.DOCX]
